# Hidden lattice instabilities as origin of the conductive interface between insulating LaAlO_3_ and SrTiO_3_

**DOI:** 10.1038/ncomms12773

**Published:** 2016-09-14

**Authors:** P. W. Lee, V. N. Singh, G. Y. Guo, H.-J. Liu, J.-C. Lin, Y.-H. Chu, C. H. Chen, M.-W. Chu

**Affiliations:** 1 Department of Physics, National Taiwan University, Taipei 106, Taiwan; 2 Center for Condensed Matter Sciences, National Taiwan University, Taipei 106, Taiwan; 3 Institute of Atomic and Molecular Sciences, Academia Sinica, Taipei 106, Taiwan; 4 Physics Division, National Center for Theoretical Sciences, Hsinchu 300, Taiwan; 5 Department of Materials Science and Engineering, National Chiao Tung University, Hsinchu 300, Taiwan; 6 Institute of Physics, Academia Sinica, Taipei 105, Taiwan; 7 Department of Physics and Astronomy, Rutgers University, Piscataway, New Jersey 08854, USA

## Abstract

The metallic interface between insulating LaAlO_3_ and SrTiO_3_ opens up the field of oxide electronics. With more than a decade of researches on this heterostructure, the origin of the interfacial conductivity, however, remains unsettled. Here we resolve this long-standing puzzle by atomic-scale observation of electron-gas formation for screening hidden lattice instabilities, rejuvenated near the interface by epitaxial strain. Using atomic-resolution imaging and electron spectroscopy, the generally accepted notions of polar catastrophe and cation intermixing for the metallic interface are discounted. Instead, the conductivity onset at the critical thickness of 4-unit cell LaAlO_3_ on SrTiO_3_ substrate is accompanied with head-to-head ferroelectric-like polarizations across the interface due to strain-rejuvenated ferroelectric-like instabilities in the materials. The divergent depolarization fields of the head-to-head polarizations cast the interface into an electron reservoir, forming screening electron gas in SrTiO_3_ with LaAlO_3_ hosting complementary localized holes. The ferroelectric-like polarizations and electron–hole juxtaposition reveal the cooperative nature of metallic LaAlO_3_/SrTiO_3_.

## Abstract (web summary)

The origin of interfacial conductivity between two insulating oxides, LaAlO_3_ and SrTiO_3_, remains elusive despite a long time research. Here, Lee *et al*. report atomic-scale observation of electron-gas formation for screening hidden ferroelectric-like lattice instabilities, discounting the role of polar catastrophe and cation intermixing.

## Graphical abstract

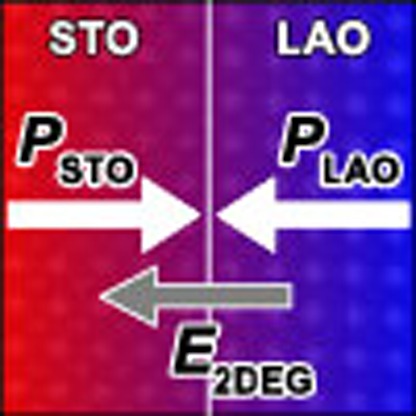

The metallic LaAlO_3_(LAO)/SrTiO_3_(STO) interface^1^ fuels the modern quest of electronics based on correlated electrons in oxides^2^ and the collective characteristics of superconductivity^3^, ferromagnetism^4^ and electromechanical sensitivity typical to insulating ferroelectrics (FEs)^5^,^6^ of this two-dimensional (2D) electron system manifest the manifold possibilities of oxide electronics. The conductive LAO/STO, therefore, forms the model system of emergent phenomena at oxide interfaces^1^,^2^,^3^,^4^,^5^,^6^,^7^,^8^,^9^,^10^,^11^,^12^,^13^,^14^. However, why this LAO/STO system can display so versatile physical properties^1^,^2^,^3^,^4^,^5^,^6^ remains an open question, with the origin of the 2D metallicity constituting the most basic problem in the context^1^,^2^,^3^,^4^,^5^,^6^,^7^,^8^,^9^,^10^,^11^,^12^,^13^,^14^.

Electrostatically, the bulk ABO_3_ perovskite, LAO, is non-polar^7^. The layer-by-layer thin-film growth of LAO with repeated AO and BO_2_ units can nonetheless turn the film polar by the charged essence of (LaO)^1+^ and (AlO_2_)^1−^ planes^7^. The STO substrate with Sr^2+^ and Ti^4+^ in LAO/STO heterostructures is conversely non-polar. To obtain conductive LAO/STO, a polar-catastrophe (TiO_2_)^0^–(LaO)^1+^ interface is an indispensable prerequisite, with the LAO surface being readily (AlO_2_)^1−^-terminated^7^. This electrostatic boundary condition implies an electric field (*E*∼236 meV Å^−1^) in LAO and the corresponding potential would diverge with increasing LAO thickness^8^. With 4 unit cells (uc) of LAO (lattice parameter, 3.79 Å) on STO and more, this diverging potential enables the LAO valence-band maximum to overcome the STO conduction-band minimum (offset, 3.2–3.3 eV)^8^. Charge transfer from LAO to STO readily becomes possible, diminishing the built-in field and resulting in conductive LAO/STO (refs 7, 8). Below 4-uc LAO, the interface shall be otherwise insulating^8^. This critical thickness of 4-uc LAO for the 2D electron gas (2DEG) formation in STO was indeed verified^8^,^9^,^12^,^13^,^14^, providing the evidence of polar catastrophe for the interfacial metallicity^7^,^8^. The possible existence of an interfacial metallic (La,Sr)TiO_3_ phase by cation intermixing^10^ and the introduction of electron–donor oxygen vacancies during growths^11^,^12^ have also been considered for conductive LAO/STO, whereas unable to capture the 4-uc dependence of the metallicity^8^,^9^,^12^,^13^,^14^. Polar catastrophe thus becomes the most celebrated notion on conductive LAO/STO (refs 4, 5, 6, 7, 8, 10, 11, 12, 13, 14) and also a guiding principle in designing oxide interfaces^15^,^16^,^17^.

Nevertheless, not every oxide interface with polar catastrophe is conductive^13^,^15^, and the observation of an inherent field in LAO of metallic heterostructures^14^,^18^ is inconsistent with polar catastrophe. It becomes compelling that one should also consider the less-emphasized lattice degree of freedom^16^,^19^,^20^ for unravelling the origin of the metallic interface. A quantitative tackling of the structural and intervening chemical and electronic parameters with atomic accuracy will, therefore, be most critical.

Here we report the investigation of this kind using scanning transmission electron microscopy (STEM) and electron energy-loss spectroscopy (EELS), establishing why the notions of polar catastrophe and cation intermixing are inconsistent with the whole picture and how the interfacial metallicity is originated from the structural aspect of hidden FE-like instabilities in LAO and STO.

## Results

### Quantitative unveiling of chemical and electronic features

**Figure 1:**
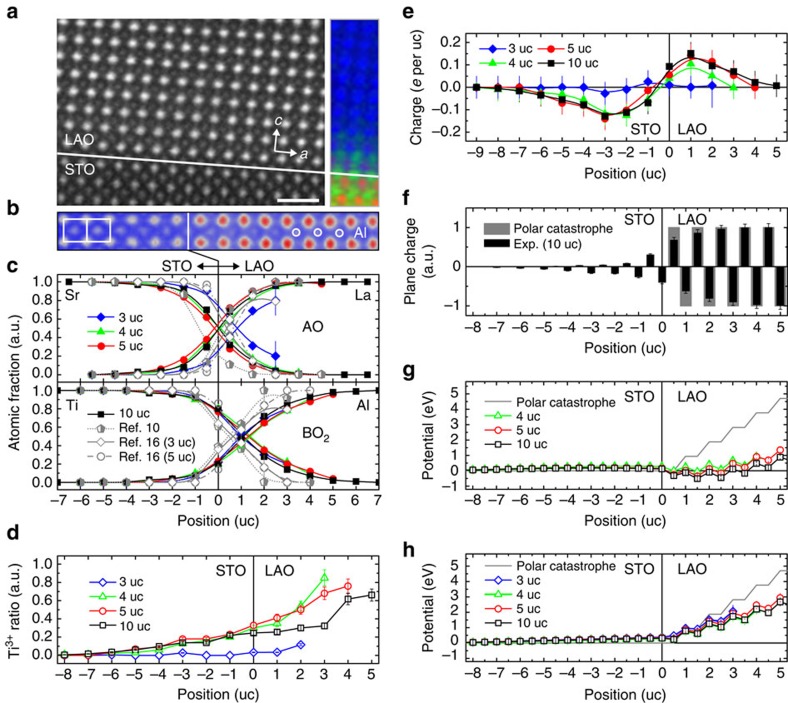
Quantitative analysis of polar catastrophe and cation intermixing in LAO/STO at atomic scale. (**a**) The summed HAADF image over three individual ones (bright, cations) and a chemical map (red, Sr; green, Ti; blue, La) in 10-uc LAO/STO. Scale bar, 1 nm. (**b**) The HAADF blowup of **a** (white and red, cations), showing the Al-site displacement towards the interface (guiding white circles) and *c*-elongated STO (guiding white rectangles). (**c**) The AO- and BO_2_-plane cation distributions, omitting the ±10% error bars for clarity of the figure presentation. The error bars on the surface LAO uc in 3-uc LAO/STO are larger, ±20%, as a result of the beam sensitivity of this uc and are particularly denoted. The SXRD results in refs 10, 16 are incorporated for comparison, while omitting the associated error bars also for clarity. The diffusive cation distribution in all these heterostructures indicates ubiquitous intermixing. (**d**) The STEM–EELS probing of the Ti^3+^ fraction (error bars, ±10%). (**e**) The uc-by-uc charge distributions derived from **c** and **d**, showing 2DEG in the STO and holes in the LAO of conductive heterostructures. Error bars, ±10% as deduced from **c** and **d** except for the larger error of ±20% in the surface uc of 3-uc LAO/STO due to the associated beam sensitivity. (**f**) The plane-specific charges in the polar-catastrophe context (grey) and in the 10-uc experiment (black) considering the uc charges in **e**. (**g**) The plane-by-plane potential variations in the polar-catastrophe model (grey) and on the presence of interfacial charges in **e**. The insulating 3-uc LAO/STO is free from charge at the interface, thus not shown. (**h**) The counterpart to **g** considering cation intermixing in **c** only without net interfacial charge (that is, charge-neutral uc). Cation intermixing grades the diverging polar-catastrophe potential and the associated interfacial-metallicity onset at 4-uc LAO is no longer satisfied.

STEM and EELS were performed on insulating 3-uc and metallic 4-, 5- and 10-uc LAO/STO, all of which were optimally post-annealed to restore oxygen vacancies (Methods). Figure 1a shows the high-angle annular dark-field (HAADF) imaging of 10-uc LAO/STO, which is the sum over three images for ensuring good representativeness. The coloured panel in Fig. 1a is an individual STEM–EELS chemical map. Figure 1b exhibits the HAADF blowup, unravelling Al-site displacements towards the interface and tetragonality in STO. Figure 1c is the averaged chemical profiles over five STEM–EELS maps, acquired on different regions of each LAO/STO with small deviation among the five corresponding data sets (Supplementary Fig. 1), and thus-statistically derived results suggest AO- (La and Sr) and BO_2_-plane (Al and Ti) cation concentrations^15^,^21^. The diffusive cation distribution at the interface (Fig. 1c) indicates intermixing^10^,^15^,^16^,^21^ and appears not only in insulating 3-uc, but all conductive LAO/STO (grey, known intermixing in insulating 3-uc and metallic 5-uc heterostructures by surface X-ray diffraction^10^,^16^, SXRD). Figure 1d shows the STEM–EELS electronic probing of Ti (the only mixed-valence element rendering its charge-accommodation role), averaged over five data sets. Summing up Fig. 1c,d leads to the unprecedented uc-by-uc charge distributions in LAO/STO (Fig. 1e; Methods), with 2DEG in the STO of metallic heterostructures and, surprisingly, holes in the LAO (ref. 22). The insulating 3-uc LAO/STO is free from interfacial charge.

The plane-specific charge deduced from Fig. 1e is shown in Fig. 1f (for clarity, 10 uc only) and leads to the plane-by-plane potential variations in Fig. 1g according to the Gauss law in Maxwell's equations^8^,^13^. Figure 1h displays the intermixing counterparts (charge-neutral uc without interfacial charges) of the potential variations. Notably, cation intermixing (Fig. 1h) reduces the diverging potential of polar catastrophe (grey), like the graded electrostatic divergence in polar semiconductor heterojunctions by intermixing^23^. Polar catastrophe no longer addresses the 4-uc dependence of the metallicity^8^,^9^,^12^,^13^,^14^ (Fig. 1h). The increasing 2DEG density with increasing LAO thickness, expected within the framework of the diverging polar-catastrophe potential^8^,^14^, is also discounted by the nearly constant 2DEG in metallic LAO/STO (Fig. 1e). The ubiquitous intermixing in both insulating and conductive LAO/STO in Fig. 1c is evidently for grading polar catastrophe^15^,^23^, and this intermixing tendency, characteristic to heterostructural growths^10^ (Methods; Supplementary Fig. 2), is indeed general in the different STEM–EELS and SXRD characterizations of various LAO/STO specimens (Fig. 1c)^10^,^16^. Polar catastrophe and cation intermixing do not dictate the interfacial metallicity.

### Picometre-scale characterization of FE-like distortions

**Figure 2:**
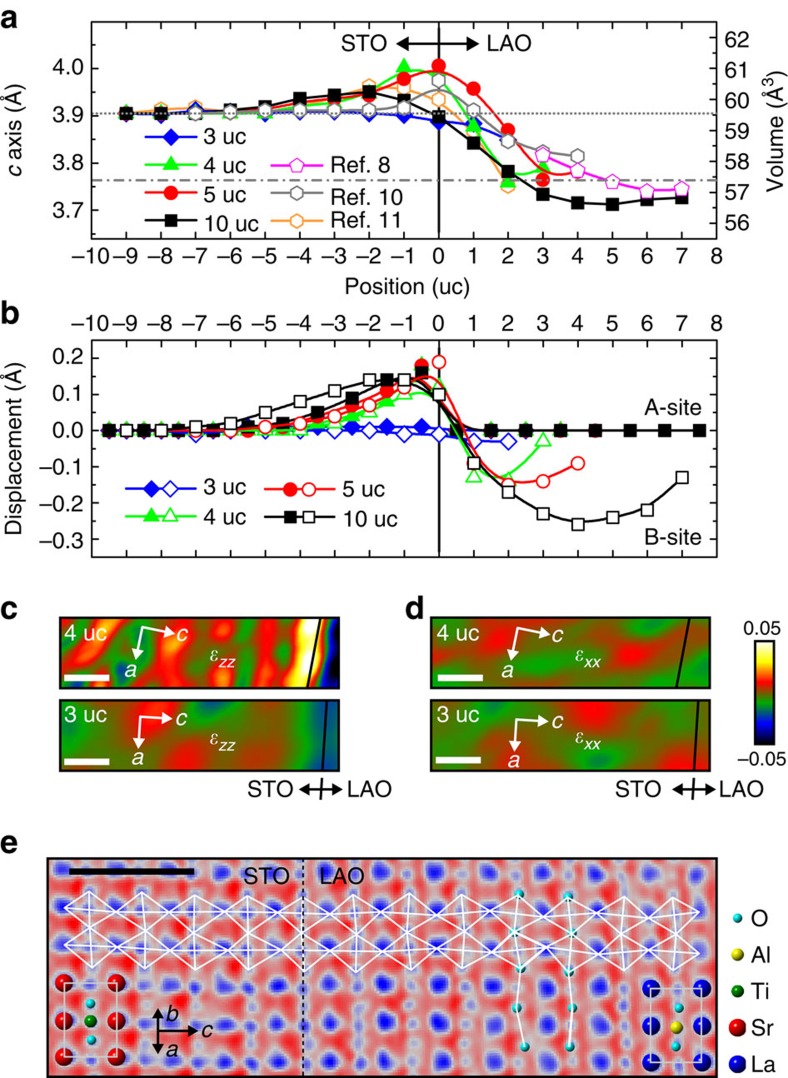
Structural distortions and FE-like symmetry breaking in LAO/STO heterostructures. (**a**) The *c* axis variations and uc-volume changes in LAO/STO. The SXRD results of the heterostructures with various LAO thicknesses^8^ and in metallic 5-uc LAO/STO (refs 10, 11) are incorporated for comparison (omitting the associated error bars for clarity of the figure presentation). Grey dotted (dotted–dashed) line, 3.905 (3.762) Å of bulk STO (pseudomorphic-strained LAO). (**b**) The atomic displacements corresponding to **a**. Positive sign directs to the LAO surface. See text for detail. Each point in **a** and **b** represents the averaging over 150 separate analyses and this rigorous approach ensures a structural-evaluation precision of ∼0.02 Å. The thus-estimated error bars in **a** and **b**, ±0.02 Å, are omitted for clarity. (**c**,**d**) The respective PPA-determined *ɛ*_*zz*_ and *ɛ*_*xx*_ strain maps of insulating 3-uc and conductive 4-uc LAO/STO, showing the strain characteristics basically along *c* and *a* axes. The *c* axis elongation of interfacial STO uc shows an abrupt onset on the interfacial metallicity, 4 uc in **c**. The *ab*-plane of LAO is clamped by that of STO in both insulating and metallic heterostructure (**d**). Each HAADF image for the strain mapping is the sum of three separate ones. Colour scale bar, ±5% of strain with reference to a STO area far from the interface (out of the displayed range, 16 nm × 4 nm). (**e**) The colour-coded filtered annular bright-field imaging of the characteristic anti-phase oxygen-cage rotation (guiding white cages) in 10-uc LAO/STO, revealed along [110] projection. Guiding grey frames, uc. Scale bars in **c**–**e**, 1 nm.

We now turn to the structural distortions in Fig. 1b. The lattice degree of freedom was scrutinized by peak-pair analysis (PPA) of summed HAADF images, like Fig. 1a. This image-summation method can improve the statistics of observed atomic features, and the subsequent statistical PPA characterization at sub-pixel level led to a structural-evaluation precision of ∼2 pm (Fig. 2a,b, Supplementary Fig. 3 and Supplementary Note 1)^24^. Figure 2a shows the uc-dependent variations in *c* axis (dotted line, 3.905 Å of bulk STO), and Fig. 2b exhibits the corresponding atomic displacements along *c*-direction, with solid (empty) symbols for the A-site (B-) Sr and La (Ti and Al). Figure 2c,d depict the PPA-determined strain maps of insulating 3-uc and metallic 4-uc LAO/STO for respective *ɛ*_*zz*_ and *ɛ*_*xx*_, which reveal the strain characteristics basically along *c* and *a* axes. Figure 2e displays a colour-coded annular bright-field exemplification of the cross-interface rotation of oxygen cages in 10-uc LAO/STO (Supplementary Fig. 4a for the original grey-scale image; Supplementary Notes 2 and 3).

In Fig. 2d, the similar contrast level in LAO to that in STO reveals the clamping of *ab*-plane in LAO to that in STO (2.9% tensile strain to LAO)^8^, signifying conventional pseudomorphic-strained LAO (refs 8, 25). The *c* axis should be readily contracted to 3.762 Å (dotted–dashed line, Fig. 2a; Poisson's ratio^8^,^16^, 0.24). Surprisingly, we observe gradually *c*-elongated LAO in metallic LAO/STO towards the interface (Fig. 2a), with the same tendency for the STO uc on the interfacial conductivity onset^11^,^19^ (Fig. 2c). Similar *c*-elongation characteristics were observed in otherwise SXRD (refs 8, 10, 11), TEM (ref. 26) and STEM–HAADF (refs 27, 28) investigations. The metallic heterostructures have then to be described by *c*-elongated, volume-expanded interfacial LAO and STO uc. By contrast, the insulating 3-uc LAO/STO consists of bulk-like STO and *c*-elongated LAO (Fig. 2a). This structural difference between insulating and conductive LAO/STO is further elaborated in Fig. 2b, where the positive sign points to the LAO surface. The Sr- and Ti-site displacements in Fig. 2b were determined by the displacements from the respective reference planes in undistorted, parent cubic interfacial uc^10^,^11^,^16^. The La cage in each LAO uc, instead, defines the lattice frame (thus null, Fig. 2b) for the precise determination of the Al-site off-centre displacement (like Supplementary Fig. 3), which is the central structural character of the LAO (ref. 16). The high-precision, statistical determination of atomic displacements in LAO/STO (Fig. 2b) was not reported in the previous TEM and STEM–HAADF studies^26^,^27^,^28^. All displacements in conductive LAO/STO point to the interface (Fig. 2b), breaking the centrosymmetry of bulk LAO and STO like FEs^11^,^19^,^27^ (Table 1 and Supplementary Note 4), whereas the insulating 3-uc counterpart preserves the cubic essence with negligible off-centre atomic displacements.

The metallic heterostructures are firmly addressed by FE-like symmetry breaking, while the insulating LAO/STO shows *c*-elongated, symmetry-preserved LAO. All these structural features are unexpected considering a typical pseudomorphic epitaxy under the tensile strain should lead to uniformly *c*-contracted LAO without symmetry breaking^8^,^13^,^25^. It naturally raises the question: what if the epitaxial strain is accommodated by FE-like distortions?

**Figure 3:**
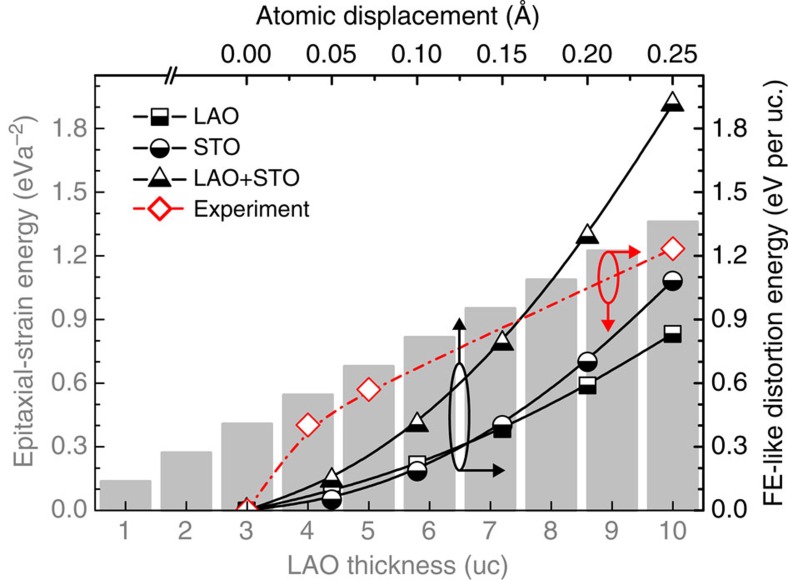
FE-like distortions in LAO and STO as a strain-accommodation alternative. Grey histograms, the evolution of the epitaxial-strain energy with increasing LAO-film thickness. Black curves, the FE-like distortion energies in LAO and STO as a function of the symmetry-breaking atomic displacements (*d*_A,B_). Red dotted–dashed line, the respective FE-like distortion energies in 4-, 5- and 10-uc LAO/STO contributed by the maximal atomic displacements, *d*_A,B_, in the interfacial LAO and STO uc. The insulating 3-uc counterpart is free from FE-like distortion, thus null. All calculations ignore cation intermixing for simplicity. Above 3 uc, the evolution of the FE-like distortion energy (red) closely mimics that of the epitaxial-strain energy, providing the energetics footing of the FE-like distortions as an alternative for strain accommodation. See text for detail.

We readily evaluate the corresponding epitaxial-strain and FE-like distortion energies^16^,^29^ (Supplementary Note 5), with the former representing the cost of the *c*-contracted LAO and scaling with ∼0.136*t* eV*a*^−2^ (*t*, LAO thickness in uc; *a*, 4 Å for convenience). The latter involves the atomic-buckling and corresponding electrostatic energies, which are the functions of A- and B-site atomic displacements from the equilibrium positions in high-symmetry reference lattices (*d*_A,B_; Supplementary Note 5)^16^. Note that the oxygen contribution to FE-like distortions is cancelled out by the anti-phase oxygen-cage rotation^30^ (Fig. 2e and Supplementary Fig. 4) and Fig. 2b signifies *d*_A,B_. In Fig. 3, we show the associated epitaxial-strain (grey histograms) and FE-like distortion energies (black curves). The red dotted–dashed curve (Fig. 3) specifically indicates the FE-like distortion energies in respective 4-, 5- and 10-uc LAO/STO due to the maximal off-centre Al-site (Ti- and Sr-site) displacements of ∼0.1 (both ∼0.1), ∼0.15 (∼0.1) and ∼0.25 (∼0.15) Å in the interfacial LAO (STO) uc in Fig. 2b. The insulating 3-uc counterpart is free from FE-like distortion, thus null in Fig. 3 (red dotted–dashed).

Notably, the evolution of the epitaxial-strain and FE-like distortion energies above 3-uc LAO closely mimics each other (Fig. 3), implying comparable costs between the pseudomorphically *c*-contracted LAO and the FE-like distorted LAO and STO, and establishing the energetics footing of the FE-like distortions as a strain-accommodation alternative. The close similarity of elastic properties of LAO (ref. 31) and STO (ref. 32) should be critical, since the misfit-strain field would readily distort both the materials rather than, in principle, the film^25^. This requirement of simultaneously strained LAO and STO finds a viable solution in the hidden FE-like instabilities of the materials, which are resurrected by stretching the uc (Fig. 2a,b) as also microscopically suggested in our *ab initio* calculations (Supplementary Figs 5 and 6 and Supplementary Notes 2 and 3). The *c*-elongated LAO in insulating 3-uc LAO/STO, therefore, indicates a tendency towards this FE-like state and the corresponding accumulated epitaxial-strain energy should be close to that required for resurrecting the hidden FE-like instabilities in LAO/STO.

### Head-to-head polarizations across the metallic interface

**Figure 4:**
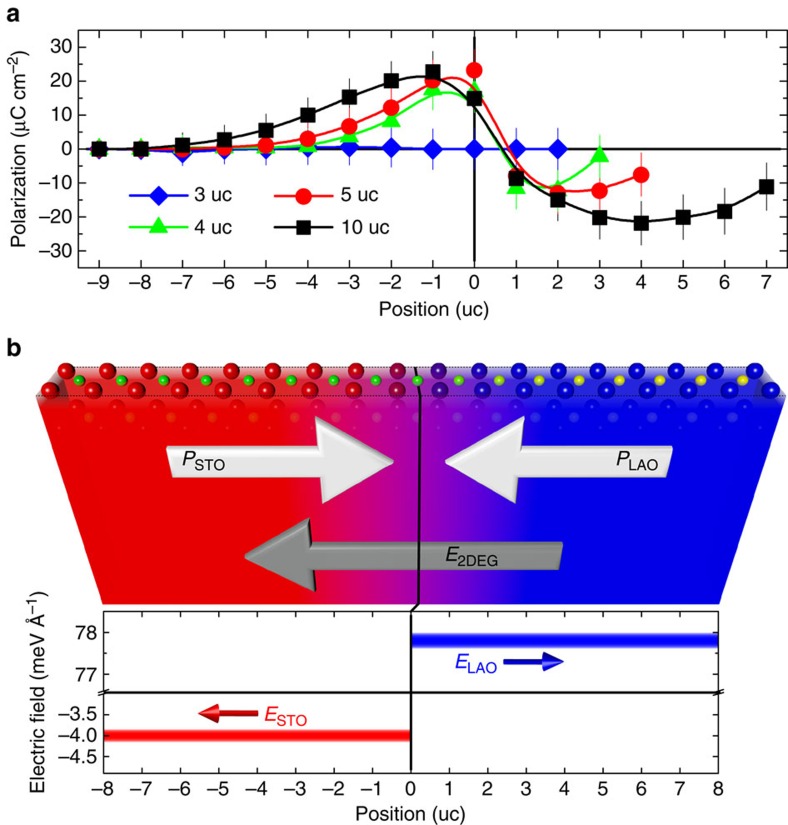
The strain-resurrected head-to-head FE-like polarizations and the 2DEG-hole juxtaposition in conductive LAO/STO. (**a**) The formation of head-to-head polarizations, *P*_LAO_ and *P*_STO_ in **b**, across the metallic LAO/STO interfaces, while null polarization for the insulating 3-uc counterpart. Positive sign points to the LAO surface. (**b**) The field associated with the juxtaposed 2DEG-hole, *E*_2DEG_, in the 10-uc exemplification along with the respective depolarization fields, *E*_LAO_ and *E*_STO_, of *P*_LAO_ and *P*_STO_. The diverging potentials corresponding to *E*_LAO_ and *E*_STO_ drive the 2DEG formation, and the complementary holes are localized. Scheme, the geometry of *P*_LAO_, *P*_STO_, and *E*_2DEG_ with the atomistic structure of the contributing LAO and STO uc (top) derived from Fig. 2a,b, and the artwork colour coding following the associated cation intermixing in Fig. 1c. The 2DEG in STO perfectly screens *E*_STO_. The anti-parallel *E*_2DEG_ to *E*_LAO_ is nonetheless like poling *P*_LAO_, with the accompanied *E*_LAO_ suggesting a FE-like origin for the observed field in the LAO (Fig. 1g; refs 14, 18).

Figure 4a shows the FE-like polarizations in all interfacial uc, *P*=*q*_A,B_*d*_A,B_/*V* (*P*, polarization; *q*_A,B_, the cation valence in Fig. 1e; *d*_A,B_, Fig. 2b; *V*, uc volume in Fig. 2a) in the point-charge approximation^29^,^33^. While the insulating LAO/STO is free from polarization, the metallic heterostructures show head-to-head polarizations across the interface (Fig. 4b), strikingly similar to the head-to-head, charged domain walls (DWs) in insulating FEs^29^,^33^.

Within the framework of Maxwell's equations, the divergent Coulomb repulsion of the head-to-head polarizations set-ups a pair of depolarization fields, which symmetrically point away from the DWs (for example, Fig. 4b), and the corresponding diverging-potential pair (analogous to polar catastrophe) renders the DWs as electron reservoirs. The thus-accumulated electron gas screens the polarizations and turns the DWs charged and conductive, with the electron-gas density (*n*) of *n*=2 *P*/*ew* (*w*, DW width)^29^,^33^. Maxwell's equations are universal and the head-to-head polarizations in the 4-, 5- and 10-uc LAO/STO (Fig. 4a) should dictate the 2DEG formation, with *n*=(*P*_LAO_+*P*_STO_)/*ew* (*w*, the contributing LAO and STO uc). In 10-uc LAO/STO (Fig. 4a and Table 1), the respective polarizations of contributing LAO and STO are *P*_LAO_ ∼16.5 and *P*_STO_ ∼11.6 μC cm^−2^ in average, referring to *n* ∼2.7 × 10^20^ cm^−3^≈∼4.2 × 10^13^ cm^−2^ that is in remarkable agreement with the average 2DEG in STO (∼4.4 × 10^13^ cm^−2^; Fig. 1e) and the corresponding Hall measurement (∼4.6 × 10^13^ cm^−2^; Table 1). The average density of holes in the LAO, ∼4.6 × 10^13^ cm^−2^ (Fig. 1e), is surprisingly close to that of 2DEG in the STO, directing to an overall charge balance in the context of classical electrostatics^20^,^22^. The metallic 4- and 5-uc LAO/STO show comparable quantitative agreements (Table 1). The interfacial conductivity in LAO/STO is firmly mandated by the strain-resurrected head-to-head polarizations. A sublte difference in the maximum-*P*_STO_ location (#–1 STO uc for 10-uc; #–1–0 uc for 4- and 5-uc LAO/STO; Fig. 4a) may be related to the different length scales involved in the strain accommodation (*w*, Table 1).

Considering the holes in LAO, it was suggested that the formation of 2DEG in the STO of conductive LAO/STO should be accompanied with complementary holes in the LAO in view of an electrostatic balance^20^,^22^. This possible presence of holes^22^ is, however, not particularly noticed, because Hall measurements of the metallic LAO/STO reveal electron contributions only^8^,^9^,^13^,^22^. The holes therein would be localized due probably to the subtle structural disorders associated with LAO (ref. 22) and the corresponding hole probing requires techniques beyond typical transport measurements, with EELS, which tackles unoccupied density of states above Fermi level and is sensitive to electron and hole doping at uc scale^34^, being an ideal tool. The holes observed in LAO of metallic 4-, 5- and 10-uc LAO/STO (Fig. 1e) could readily be attributed to the complementary holes to the 2DEG in STO, instead of FE-like polarization bound charges that should otherwise appear in both LAO and STO. Indeed, FE polarizations comprise polarized lattice dipoles and do not involve a net change in charge density (that is, mixed-valence features) on either side of the polarizations^35^,^36^,^37^. Take FE Ba^2+^Ti^4+^O_3_, for example, the associated EELS studies did not reveal a change of Ti-valence state of nominal 4+ throughout the material^36^,^37^. An essence of FE-like bound charges for the holes in LAO (Fig. 1e) can be excluded^35^,^36^,^37^, and the presence of the holes only in LAO further supports their complementary character to the 2DEG in STO (ref. 22).

## Discussion

The 2DEG-hole juxtaposition in the LAO/STO is similar to a plate capacitor^38^ with *E*_2DEG_≈∼4.5 meV Å^−1^ (Fig. 4b, 10 uc) and the otherwise FE-like depolarization fields of *P*_LAO_ and *P*_STO_ are *E*_LAO_ ∼77.8 and *E*_STO_ ∼4.0 meV Å^−1^ (Table 1), respectively. Indeed, 2DEG perfectly screens *P*_STO_ (Figs 1e and 4) and, therefore, *E*_STO_ is diminished^29^,^33^,^38^, consistent with the vanishing potential in Fig. 1g. The anti-parallel *E*_2DEG_ to *E*_LAO_ is nonetheless like poling *P*_LAO_ (Fig. 4b), mediating robust *E*_LAO_ that could account for the observed field in the LAO of metallic LAO/STO (Fig. 1g; 80.1 for 20 uc, ref. 14; ∼30 meV Å^−1^ for 5 uc, ref. 18). Moreover, the LAO in conductive heterostructures tends to restore to the *c*-contracted, symmetry-preserved limit (grey dotted–dashed in Fig. 2a; and Fig. 2b) from the eighth uc, with the same observation in heterojunctions thicker than 8-uc LAO (ref. 8). Our investigations thus propose an upper bound of eight respective LAO and STO (ref. 19) uc, which display the strain-rejuvenated head-to-head polarizations, and the LAO (STO) uc atop (beneath) are bulk-like, centrosymmetric, corroborating the finite length scale of an interfacial strain field^25^.

Indeed, there have been attempts to address the polarization distortions in LAO/STO using SXRD (refs 11, 16) and HAADF (ref. 27), and these experimental reports are basically related to the theoretical prediction of a FE-like dipole in the LAO of insulating LAO/STO, pointing to the LAO surface to compensate for the corresponding polar-catastrophe electric field^20^. In metallic LAO/STO, the otherwise vanishing field in the LAO is accompanied with diminishing FE-like distortions^20^. While the SXRD report of ref. 16 supports the theoretically suggested emergence and disappearance of polarizations in the LAO as a function of the interfacial metallicity^20^, ref. 11 unveiled that, in metallic LAO/STO, the structural degree of freedom at play should rather reside in the STO. The HAADF study^27^ proposed a separate pattern, with the FE-like polarization in LAO (STO) pointing to (away from) the metallic interface. These experimental contributions^11^,^16^,^27^ show conflicting structural conclusions, and the theoretical report^20^ has not taken into account the relevance of structural distortions in STO, which were nonetheless established in Fig. 2a and various reports^10^,^11^,^19^,^26^,^27^,^28^,^39^. A close examination of refs 11, 16, 27 reveals that the characteristic anti-phase oxygen-octahedral rotations in LAO/STO (*a*^−^*a*^−^*c*^0^-type in Glazer's notation^30^, Fig. 2e and Supplementary Fig. 4) have not been noticed therein. The improper reference-LAO lattice used in the SXRD deduction of atomic displacements^11^,^16^, and the compromised electron-optics condition used in the HAADF imaging^27^ lead to further inconsistencies on the characterized structural details^11^,^16^,^27^. These dissatisfactory factors in the experimental^11^,^16^,^27^ and theorical^20^ studies may account for why our unambiguous observation of head-to-head FE-like polarizations (Fig. 4) was not found therein.

There exists another theoretical report concerned with polarizations in LAO/STO (ref. 40) in addition to ref. 20, while neither works predicted head-to-head polarizations in conductive heterostructures. Indeed, both reports could not have been aware of the three experimentally observed structural factors hereby, simultaneously strained interfacial LAO and STO uc due to the similar elastic constants of the materials, hidden FE-like instabilities of the strained LAO and STO, and accompanied octahedral rotations. The lack of considerations on these lattice degrees of freedom^20^,^40^ may explain why there is no existing theoretical prediction on our experimentally resolved polarization configuration. It is nonetheless noted that head-to-head polarizations are customarily found in *n*-type FEs, such as BaTiO_3_ (ref. 41) and (Ca,Sr)_3_Ti_2_O_7_ (ref. 42), whereas the tail-to-tail counterparts prefer *p*-type FE materials, for example, HoMnO_3_ (ref. 43) and YMnO_3_ (ref. 44). This intriguing correlation is addressed by the conventional electrostatic argument that head-to-head (tail-to-tail) polarizations are to be screened by electrons (holes)^29^. Concerning LAO/STO, STO is known to be *n*-type, while LAO features the Fermi level at mid-gap^14^. Electrons readily constitute the most apparent carriers in LAO/STO and head-to-head polarizations may thus be favoured in the heterostructures. This proposed electrostatic motif for the polarization geometry would, however, need to be scrutinized by future first-principles and phenomenological mean-field calculations that can incorporate the three structural ingredients.

Our exploration of the interfacial conductivity in LAO/STO due to strain-rejuvenated FE-like instabilities yields broad implications to the metallic interfaces in ZnO/ZnMgO (ref. 45) and AlGaN/GaN (ref. 46), where residual piezoelectric moments appear across the interfaces of these wide-band-gap materials. What in common in these oxide and semiconductor heterojunctions is readily the presence of free charge carriers to screen the interfacial dipoles, which is an elementary concept in Maxwell's equations^40^. This prevailing cause of interfacial conductivity in accordance with Maxwell's equations provides a congenial picture for future integration of oxide- and semiconductor-heterostructure physics. We believe that the FE-like sensitivity of conductive LAO/STO to electromechanical stimuli^5^,^6^ can find a structural basis herein, since the suggested contribution by electron–donor oxygen vacancies in the LAO (refs 5, 6) is inconsistent with the holes found in the film (Fig. 1e). The current discovery also provides new structural hints for future experimental, theoretical investigations of the origin of superconductivity^3^ and ferromagnetism^4^ in metallic LAO/STO at low temperatures. Our quantitative atomic-scale investigations advance the understanding in LAO/STO and would stimulate explorations of exotic strained 2D states like strain-resurrected magnetic, topological order parameters at crystallite boundaries^47^,^48^.

## Methods

### The growth of LAO/STO heterostructures

The pulsed-laser depositions of (001)-oriented LAO/STO heterostructures were performed on TiO_2_-terminated substrates at 850 °C under the oxygen pressure of 2 × 10^−5^ torr, with the post-annealing at 700 °C for 20 min under 500 torr oxygen^18^. These growth and oxygen post-annealing conditions are compatible with the suggested parameters for optimally reducing oxygen-vacancy contributions to the interfacial metallicity^49^ (Supplementary Note 6). The STEM and EELS studies were performed on thus-grown LAO/STO with 3-, 4-, 5- and 10-uc LAO. The 4-, 5- and 10-uc (3-uc) LAO/STO are metallic (insulating) as expected^9^, and the Hall measurements revealed the carrier density, 3∼6 × 10^13^ cm^−2^ (Table 1), characteristic of conductive heterostructures^8^,^9^,^49^. The 2DEG density has been known to show little variation as a function of temperature and only gently rises below 30 K, with the low-temperature upturn in electron density to be associated with increased electrostatic screening by the large dielectric constant of STO at low temperature^50^. While STO is subject to a tetragonal phase transition at ∼105 K, this low-temperature phase is centrosymmetric (space group, *I*4*/mcm*) and constitutes the ground state of STO (refs 51, 52), with noticeably small tetragonal distortion of *c*_p_/*a*_p_ ∼1.001 (p, primitive cell) and free from polarization displacement^52^. These two latter structural ingredients of STO at low temperature would, therefore, only marginally affect the head-to-head polarizations formed at room temperature, and the effect of increased dielectric constant of STO at low temperature is basically on the screening characteristics of 2DEG (refs 50, 53). The 2DEG densities in Table 1 represent the characteristic values at 80 K.

### The STEM imaging and STEM–EELS chemical mapping

The STEM investigations were conducted on a JEOL-2100F microscope, operated at 200 keV and equipped with a CEOS spherical-aberration corrector and a Gatan-Efina EELS spectrometer^15^,^21^,^54^. The cross-sectional samples were prepared by conventional mechanical polishing, followed by Ar-ion milling and a gentle Ar^+^-beam cleaning before the STEM studies^15^,^21^. The thickness of the specimens along beam incidence is systematically 0.3–0.4 *λ* (*λ*, the inelastic mean free path) and a probe current of ∼78 pA was exploited. The HAADF and annular bright-field imaging experiments were subject to the respective collection angles of 72–192 and 7.2–19.2 mrad, with the frame time of ∼8 s (32 μs per pixel; 512 × 512 pixels; 0.36 Å per pixel). The STEM–EELS chemical mapping was performed using a pixel time of 150 ms and the line-scan tackling of fine Ti *L*- (Supplementary Fig. 7a) and O *K*-edge spectra (Supplementary Fig. 8a) was conducted with an exposure time of 600 ms per pixel and a dispersion of 0.2 eV per pixel (corresponding energy resolution, ∼1.0 eV). All STEM–EELS results were acquired on an EELS collection angle of 30 mrad and subject to careful noise reductions by principle component analysis^15^,^21^. To be more specific about the effect of oxygen post-annealing on relevant vacancy healing, control STEM–EELS experiments were performed on 10-uc LAO/STO without post-annealing, which exhibits a higher electron density of ∼1.1 × 10^14^ cm^−2^ (Hall measurements, 80 K) as expected for metallic LAO/STO of this kind with additional electron contributions by oxygen vacancies^49^. The STEM–EELS results (Supplementary Fig. 8) indeed revealed traces of oxygen vacancies in the LAO/STO without post-annealing, while the 3-, 4-, 5- and 10-uc LAO/STO with post-annealing, studied throughout Figs 1 and 2, are relatively stoichiometric in oxygen content (Supplementary Note 6). For the STEM–EELS mapping, La-*M*_4_, Sr-*M*_3_ and Ti-*L*_2_ edges were used for obtaining each average elemental profile^15^,^21^ (La, Sr and Ti) across the 3-, 4-, 5- and 10-uc heterostructures in Fig. 1c and Supplementary Fig. 2 (a comparison between 10-uc LAO/STO with and without post-annealing). The oxygen post-annealing does not alter the degree of cation intermixing (Supplementary Fig. 2) and the ubiquitous intermixing in Fig. 1c has already been formed on the heterostructural growths^10^. The length scales of the distorted LAO and STO uc in conductive LAO/STO (for example, both ∼8 uc in the 10-uc heterostructure, Fig. 2a) are different from those of the cation intermixing (both 4–5 uc in the 10-uc LAO/STO, Fig. 1c) and Ti^3+^ fraction (Fig. 1d). The suggested possibility of the structural distortions in metallic LAO/STO due to intermixing and/or the presence of Ti^3+^ (ref. 10) can be excluded, and correlation between oxygen vacancies and FE-like polarizations could be ignored considering negligible oxygen vacancies herein. The similarity in the intermixing length scales between the smaller B- (Al and Ti) and larger A-site cations (La and Sr) in Fig. 1c may be associated with the intermixing for grading polar catastrophe. The Ti-valence estimation in Fig. 1d is based on the least-square fitting of the EELS spectra^7^,^55^ summed over five sets of atomically resolved data (for example, Supplementary Fig. 7a,b) to ensure statistics^15^,^21^. The respective chemical maps of 3-, 4- and 5-uc LAO/STO are shown in Supplementary Fig. 1b–d. SXRD and STEM–EELS are the only two existing techniques for atomic-scale chemical quantifications, and STEM–EELS further allows an electronic probing of transition-metal valences (Supplementary Fig. 7a,b) unavailable by SXRD (refs 10, 11, 16). Nonetheless, the deep Al-*K* edge at 1,560 eV is beyond the limit of our EELS instrumentation (Supplementary Fig. 9). The Al profile was derived by the formula of (1–Ti), which is otherwise acceptable considering our high confidence level in the Ti quantification^15^,^21^ (Supplementary Note 7). The uc-by-uc charge density in Fig. 1e is derived from the net charge in each interfacial uc with a general chemical formula of (La^3+^_*x*_Sr^2+^_1–*x*_)(Al^3+^_*y*_Ti_1–*y*_)O_3_, for which the uc-dependent *x* and *y* can be estimated by Fig. 1c. Considering that each interfacial uc preserves the nominal charge neutrality, the corresponding Ti-valence state would then be (4−*x*−3*y*)/(1−*y*). An experimentally determined Ti valence, Fig. 1d, higher (lower) than this nominal valence of (4−*x*−3*y*)/(1−*y*) suggests the existence of hole (electron) in the given uc^15^,^21^. This ionic-limit principle has been known to be appropriate for LAO/STO (refs 7, 8).

### Data availability

The data that support the findings of this study are available from the corresponding author M.-W.C. on request.

## Additional information

**How to cite this article:** Lee, P. W. *et al*. Hidden lattice instabilities as origin of the conductive interface between insulating LaAlO_3_ and SrTiO_3_. *Nat. Commun.* 7:12773 doi: 10.1038/ncomms12773 (2016).

## Supplementary Material

Supplementary Information Supplementary Figures 1-9, Supplementary Notes 1-7 and Supplementary References

## Figures and Tables

**Table 1:** The electronic and structural characteristics of conductive LAO/STO.

Characteristics	4 uc	5 uc	10 uc
2DEG (cm^−2^) *	4.3 × 10^13^	4.8 × 10^13^	4.4 × 10^13^
2D holes (cm^−2^)*	4.1 × 10^13^	4.6 × 10^13^	4.6 × 10^13^
*P*_LAO_ (μC cm^−2^)*	8.7	10.2	16.5
*P*_STO_ (μC cm^−2^)*	6.0	8.4	11.6
*n* (cm^−2^)†	4.0 × 10^13^	4.1 × 10^13^	4.2 × 10^13^
*w* (uc)†	9 (6, STO; 3, LAO)	11 (6, STO; 5, LAO)	16 (8, STO; 8, LAO)
*E*_2DEG_ (meV Å^−1^)‡	4.4	4.9	4.5
*E*_LAO_ (meV Å^−1^)§	41.0	48.1	77.8
*E*_STO_ (meV Å^−1^)§	2.0	2.9	4.0
Hall measurement (cm^−2^)	6.3 × 10^13^	3.5 × 10^13^	4.6 × 10^13^
_Δ_*ξ* (meV)||	49	50	50

^*^The experimental results in Figs 1e and 4a.

^†^The estimated 2DEG through *n*=(*P*_LAO_+*P*_STO_)/*ew*, where *w* is the total length scale of contributing LAO and STO uc.

^‡^*E*_2DEG_=*ne*/*ɛɛ*_0_, with *ɛ*∼(*ɛ*_LAO_+*ɛ*_STO_)/2 in the first approximations, *n* basically the average of 2DEG and 2D holes, and *ɛ*_0_ the vacuum permittivity.

^§^*E*_LAO_=*P*_LAO_/*ɛ*_LAO_*ɛ*_0_ and *E*_STO_=*P*_STO_/*ɛ*_STO_*ɛ*_0_.

^||^_Δ_*ξ*, band bending below the Fermi level due to the 2DEG in STO (Supplementary Note 4).
