# Supplementary material for: Hidden lattice instabilities as origin of the conductive interface between insulating LaAlO3 and SrTiO3
Source: Nat Commun. 2016 Sep 14;7:12773. doi: 10.1038/ncomms12773 (PMC5027288; doi:10.1038/ncomms12773)
Supplement: Supplementary Information — Supplementary Figures 1-9, Supplementary Notes 1-7 and Supplementary References [file ncomms12773-s1.pdf]

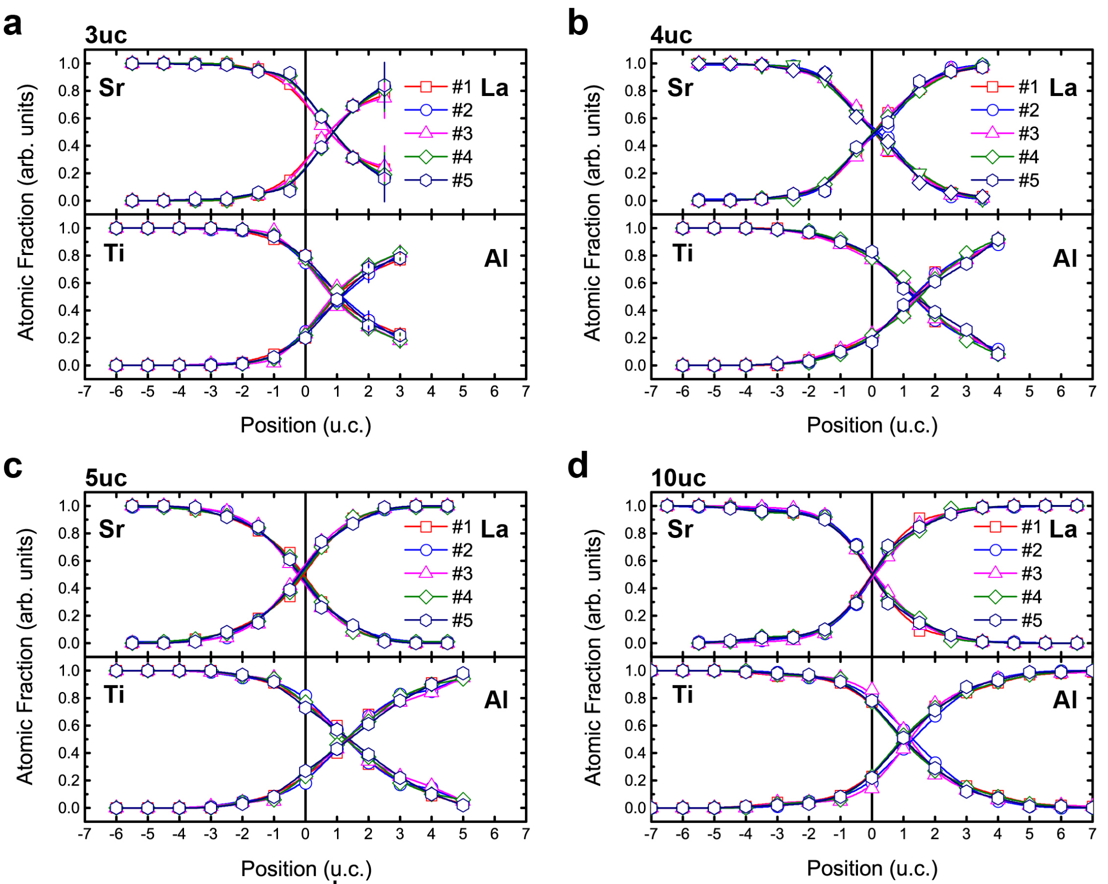


**Supplementary Figure 1│The five individual STEM-EELS datasets.** Each cation-distribution profile in Fig. 1c represents the average over five STEM-EELS datasets, which are shown in this figure (#1, 2, 3, 4, and 5) for the (**a**) 3-, (**b**) 4-, (**c**) 5-, (**d**) 10-uc LAO/STO. A small deviation among each set of data is unambiguous in (**a**)-(**d**), indicating the quality of our studies. The error bars of ± 10% are omitted for clarity of the figure presentation, except for the large value of ± 20% at the surface LAO uc in 3-uc LAO/STO due to the beam damage of this uc.

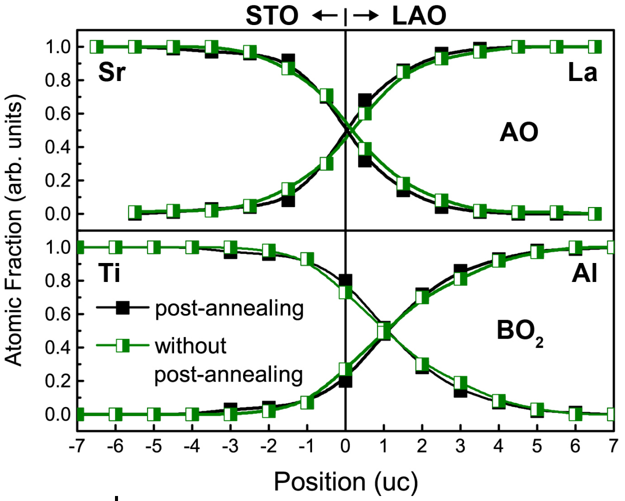


**Supplementary Figure 2│The AO and $BO_2$-plane cation distributions in 10-uc LAO/STO with and without oxygen post-annealing.** The results with post-annealing (black) were reproduced from Fig. 1c for comparison with those without post-annealing (green). Each profile represents the average over five STEM-EELS datasets and the corresponding error bars of ± 10% were omitted for clarity of the figure presentation. The similarity between the two sets of results indicates that cation intermixing has been formed upon the heterostructural growths and the post-annealing procedure does not alter the degree of intermixing.

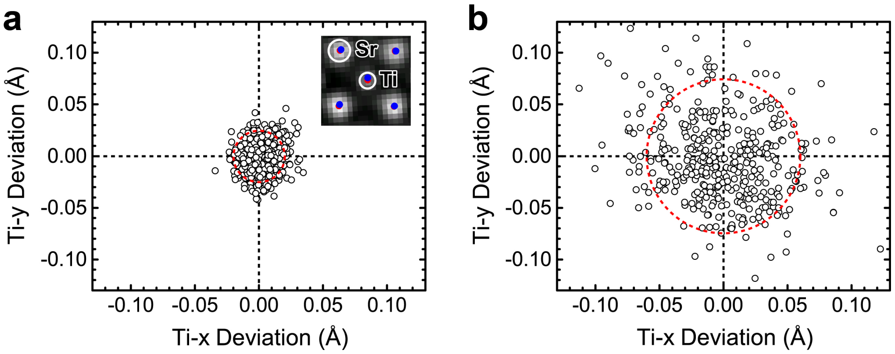


**Supplementary Figure 3│The statistical estimation of the optimal accuracy in HAADF structural characterizations using PPA. a,** The deviation of the Ti position in cubic STO uc from the ideal centrosymmetric coordinate of (0, 0) due to specimen drift and apparatus instabilities upon the HAADF imaging (inset). Inset, an uc-scale blowup of the summed HAADF image over three individual ones used for this Ti-deviation investigation. Each Ti position in (**a**) was derived from the average of the four PPA-determined, subpixel-precision coordinates of neighboring Sr atoms in a given uc (inset: red, subpixel-; blue, pixel-level determination). Pixel size, 0.36 Å. This panel (**a**) consists of analyses of this kind over ~150 uc and the standard deviation (dashed red circle) of ~0.02 Å sets the optimal accuracy of our likewise PPA characterizations conducted throughout this work. **b,** The counterpart to (**a**), while based on the PPA elaboration of a single HAADF image. The associated PPA-characterization accuracy, ~0.06 Å (dashed red circle), is notably inferior to that in (**a**) with improved statistics on the observed atomic features through the image summation. Note that the summation of more than three HAADF images did not further enhance the optimal PPA-characterization accuracy of ~0.02 Å.

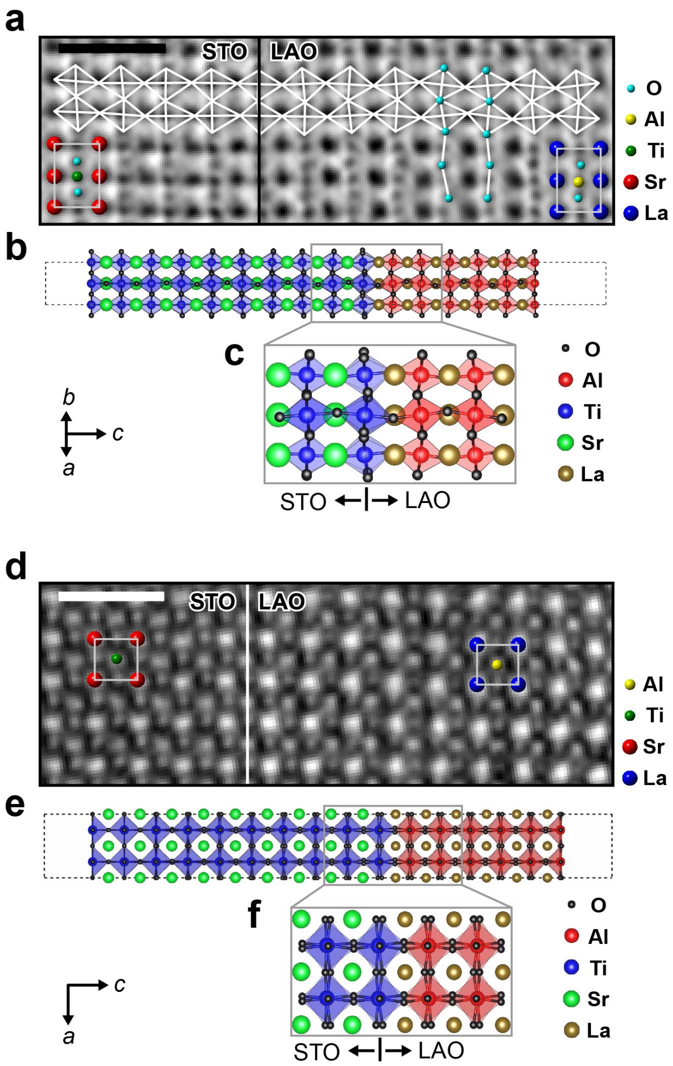


**Supplementary Figure 4│The characteristic anti-phase oxygen-cage rotations across the LAO/STO interface. a,** The filtered gray-scale ABF imaging of the anti-phase oxygen-cage rotations in 10-uc exemplification along [110] projection (the color-coded counterpart in Fig. 2e). **b,** the [110]-projected calculated oxygen-cage rotations using the supercell consisting of $(LAO)_6/(STO)_9$ and an interfacial $TiO_2$ plane. **c,** blowup of (**b**) around the interface. The characteristic anti-phase rotation pattern ($a^-a^-c^0$-type) suggests null oxygen contribution to polarization both along *c*-axis and in *ab*-plane. **d,** The contrast-inverted filtered ABF imaging of the 10-uc LAO/STO along otherwise [010] projection. **e** and **f,** the same theoretical calculations as respective (**b**) and (**c**) while projected along [010]. The characteristic anti-phase oxygen-cage rotations lead to considerable splitting of oxygen columns along [010] projection and the corresponding oxygen contrasts in the ABF image, (**d**), are readily diffused and cannot not be considered as individual oxygen columns. Scale bars in (**a**) and (**d**), 1 nm.

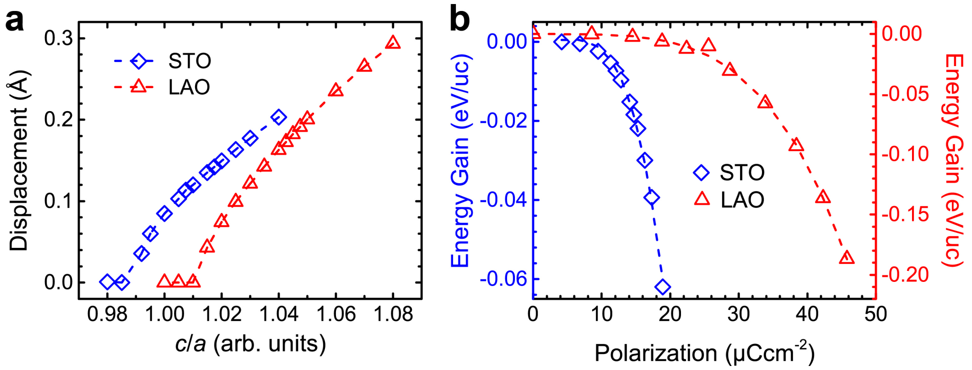


**Supplementary Figure 5│*Ab initio* calculations of FE-like polarization displacements in LAO and STO. a,** The FE-like distortions due to off-center atomic displacements in the $BO_2$ plane along *c*-axis, calculated as a function of *c/a* with $a = a_{STO}$ for both the LAO and STO. The condition of $a = a_{STO} = a_{LAO}$ for the calculated LAO signifies the stretched *ab*-plane by STO as experimentally observed. **b,** The energy gain ($\Delta E$) due to the corresponding FE-like polarizations along *c*-axis to (**a**). The AO-plane contribution is ignored for simplicity.

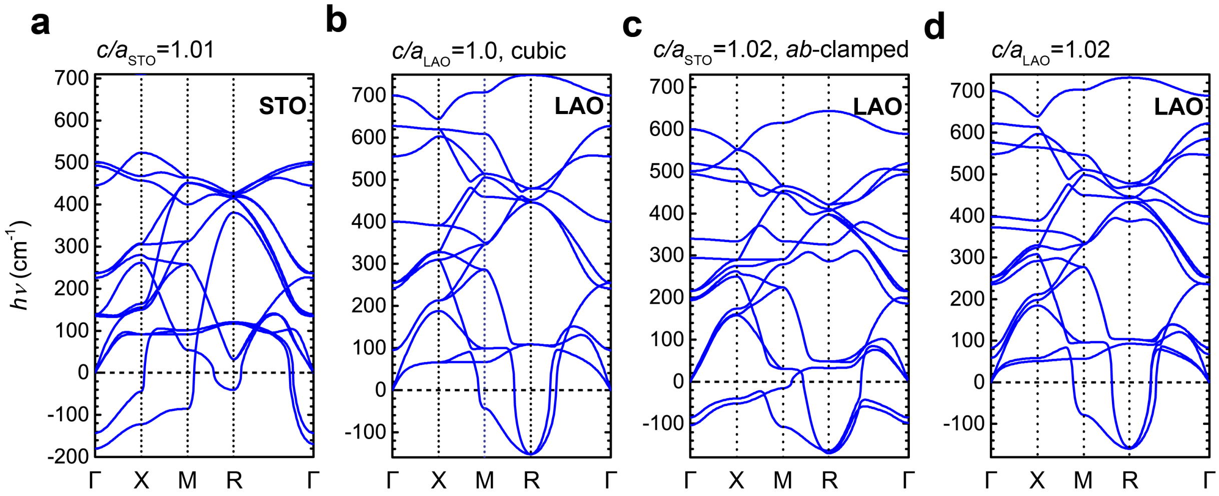


**Supplementary Figure 6│*Ab initio* calculations of the phonon dispersions in LAO and STO. a,** The phonon dispersions in *c*-elongated STO with $c/a_{STO}$ = 1.01. The soft phonon at **Γ** point is the signature of the strain-resurrected FE-like instability along *c*-axis. The negative sign of this soft phonon denotes its purely imaginary essence and reveals the lattice-instability character of this mode. **b**, The phonon dispersions in cubic LAO and (**c**) *c*-elongated LAO, $c/a$ = 1.02, with ($a = a_{STO}$) and (**d**) without the *ab*-plane clamped to that of STO. The zone-corner soft phonon at **R** point in all panels is associated with the cooperative octahedral-rotation instability in the low-temperature phases. Most importantly, the onset of the zone-center soft phonon at **Γ** point in (**c**), ascribed to the FE-like instability in the LAO along *c*-axis, can only occur when the *c*-elongation and the *ab*-plane stretching are simultaneously satisfied as experimentally suggested. The theoretically considered $c/a$ is derived from experiments.

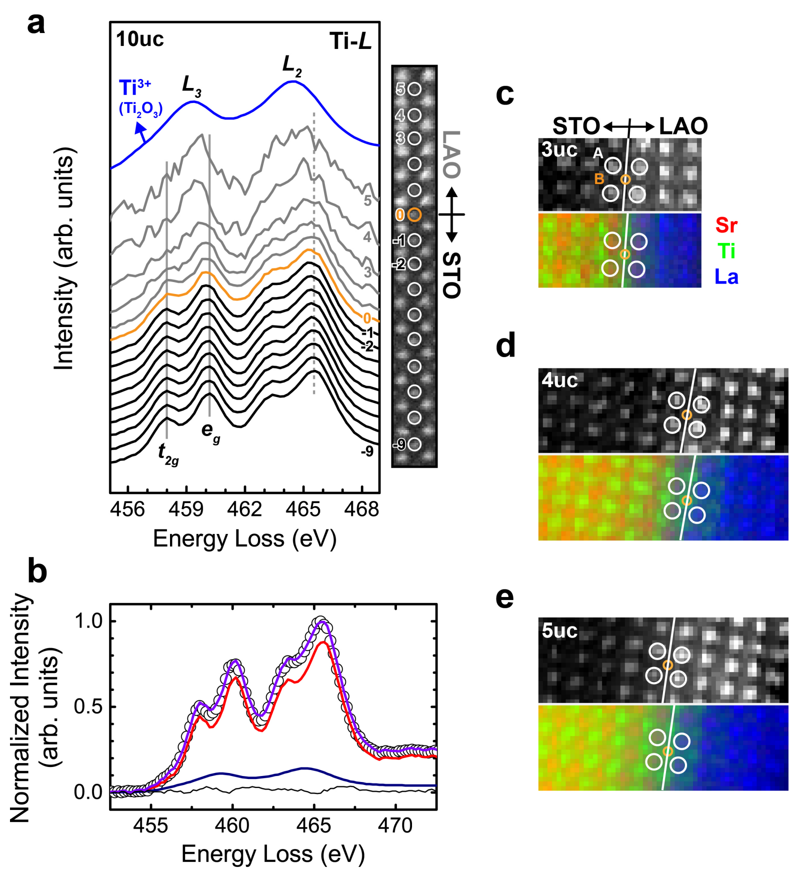


**Supplementary Figure 7│Atomically-resolved EELS spectra and STEM-EELS chemical mapping. a,** The Ti *L*-edge EELS spectra acquired on B-site columns across the interface in 10-uc heterostructure (energy resolution, ~1.0 eV; orange, interface; black, STO; gray, LAO). The HAADF image on the right indicates the atomic-column position for each spectrum shown. Each spectrum represents the sum over five separate EELS spectra acquired on different regions of the sample for ensuring statistics. The $Ti^{3+}$-reference spectrum used in the linear least-square fitting, (**b**), is also shown. **b,** The exemplification of least-squares fitting for deriving the $Ti^{3+}$ ratio, performed on the Ti spectrum at #–3 STO uc in 10-uc heterostructure. Open circles, experimental data. Purple curve, the fitted spectrum to the experimental one consisting of 13.8% of $Ti^{3+}$ (blue spectrum) and 86.2% of $Ti^{4+}$ (red reference spectrum, bare STO). Gray curve, the residual between the fitted (purple) and experimental (open-circle) spectra. The respective chemical maps (red, Sr; green, Ti, and blue, La) and the simultaneously acquired HAADF images (gray scale) in (**b**) 3-, (**c**) 4-, and (**d**) 5-uc LAO/STO.

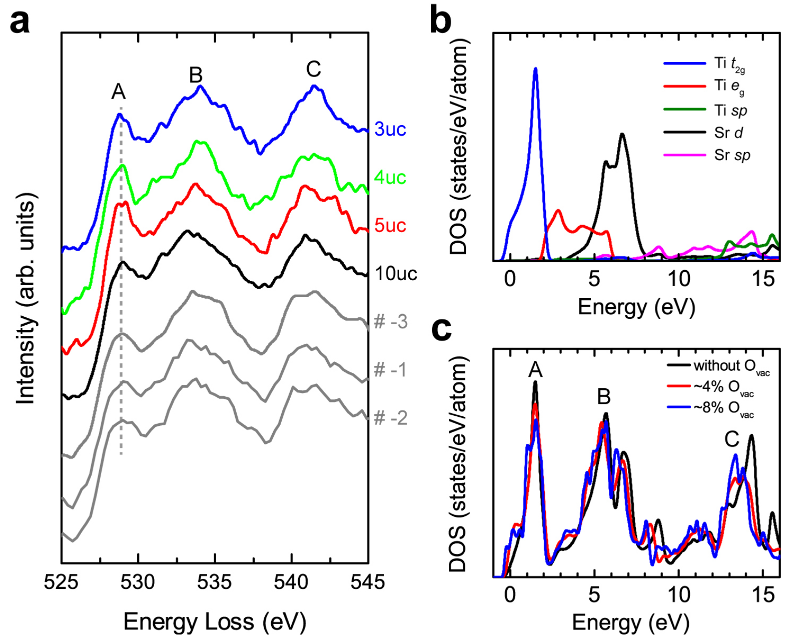


**Supplementary Figure 8│O *K*-edge EELS spectra of the STO in 10-uc LAO/STO with and without oxygen post-annealing and related theoretical calculations. a,** The O *K*-edge spectra of the STO uc #–3 in 3- (blue), 4- (green), 5- (red), and 10-uc (black) LAO/STO with oxygen post-annealing, i.e., the heterostructures investigated in Figs. 1 and 2, and those of the STO uc #–3, –1, and –2 in the 10-uc LAO/STO without oxygen post-annealing (gray). **b,** The calculated orbital-decomposed DOS of Ti and Sr in bulk STO. **c,** The calculated *p*-projected DOS of oxygen in bulk STO (without $O_{vac}$) and oxygen-deficient STO (~4 and ~8% of oxygen-vacancy $O_{vac}$). A broadening of peak A as a result of oxygen vacancies can be observed. See Supplementary Note 6 for details on peaks A, B, and C.

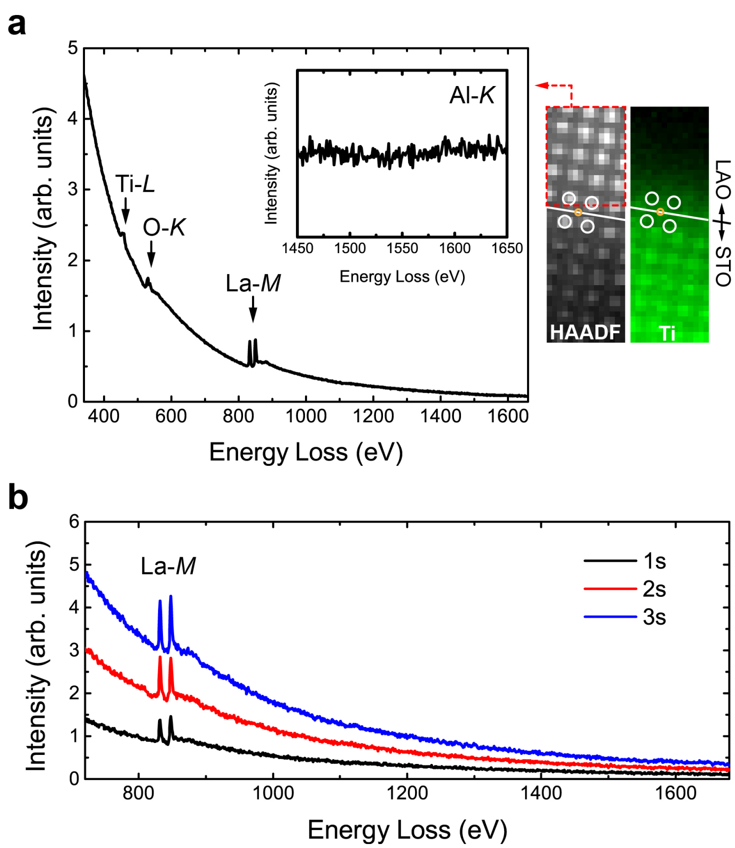


**Supplementary Figure 9│The STEM-EELS tackling of Al-*K* edge at 1560 eV in 10-uc LAO/STO. a,** The integrated STEM-EELS spectrum over the red-dashed LAO area in the simultaneously acquired HAADF image on the right (white circle, A-site cation; orange, B site). Inset, the blowup of the spectral regime of Al-*K* edge. Green panel on the right, the Ti chemical map derived from the same STEM-EELS dataset. The probe current of ~78 pA and the pixel time of 150 ms were exploited (1 eV/pixel). **b,** The EELS spectra acquired on an interfacial LAO uc with the prolonged pixel time to 1, 2, and 3 s under the same probe current and spectrometer dispersion. The Al-*K* edge is still hidden due to the low inelastic scattering cross section at the current acceleration voltage of 200 keV.

**Supplementary Note 1. EELS quality and peak-pair analysis (PPA).** The respective 5 STEM-EELS datasets used for obtaining each average chemical profile in Fig. 1c is shown in Supplementary Fig. 1. In each set of the results (Supplementary Fig. 1), small deviation can be observed, indicating the quality of our investigations. Moreover, the oxygen post-annealing does not alter the degree of cation intermixing (Supplementary Fig. 2).

Considering structural characterizations by TEM and STEM imaging, the corresponding precision would be limited by two factors[1,2], (1) pixel size (0.36 Å herein) and (2) specimen drift and apparatus instabilities upon the imaging. The PPA capturing of observed atomic features at subpixel is effective in resolving the pixel-size concern[1,3,4]. The sample drift and apparatus instabilities are then predominant in the optimal accuracy of correlated structural evaluations[1,2]. To be statistical on the estimated precision of our STEM-HAADF structural characterizations, the standard cubic uc in a bare STO-reference substrate was exploited. Using PPA, the respective positions of the four Sr and one Ti consisting of the STO uc (inset, Supplementary Fig. 3a) were captured by blue and red dots, with blue (red) signifying that the HAADF-intensity maximum corresponding to a given atom falls at a given pixel (subpixel)[3,4]. This subpixel elaboration pinpoints the observed atomic features at high accuracy[1] and was employed throughout our work to determine the coordinate, ($x$, $y$), of a given atom.

Supplementary Fig. 3a shows the Ti position averaged from the four PPA-determined coordinates of the four nearest-neighbor Sr atoms. Ideally, the centrosymmetric position of (0, 0) should be derived for the Ti. Each point in Supplementary Fig. 3a represents the deviation from this ideal position for the uc in consideration as a result of specimen drift and apparatus instabilities upon the HAADF imaging, thus posing the limit of the PPA-characterization precision[1]. Supplementary Fig. 3a is the PPA elaboration over the summation of three HAADF images into one statistically-improved image[1] (i.e., the inset) and Supplementary Fig. 3b is the control experiment on one single HAADF image, with otherwise ~150 data points for both. A standard deviation of ~0.02 Å from the ideal (0, 0) was statistically derived from Supplementary Fig. 3a (dashed red circle; ~0.06 Å, Supplementary Fig. 3b), rigorously setting the optimal precision of ~2 picometer in this STO analysis and also Figs. 2a,b that exploited the same image-summation and PPA-characterization principles. The summation of more than three HAADF images did not further enhance the structural-characterization accuracy of ~0.02 Å. The image summation in Supplementary Fig. 3a has been known to be capable of improving the statistics of observed atomic features and the correlated accuracy in structural characterizations[1]. An improved precision from ~6 to ~2 picometer is indeed observed in Supplementary Fig. 3.

Besides, the previous SXRD results of Al-site off-center displacements along $c$-axis were estimated by taking the difference between distorted LAO uc and a reference lattice frame consisting of cubic STO uc[5-7]. While this methodology is appropriate for deriving the structural detail of distorted STO (Fig. 2b)[5-7], systematic errors could exist in thus-determined Al-site displacements considering the elementary incompatibility between the reference cubic STO ($c$-axis, 3.905 Å) and typical pseudomorphic-strained LAO ($c$-axis, 3.762 Å), let alone tackling the rich $c$-axis changes and associated atomic displacements in the LAO (Fig. 2a) by the STO. The otherwise HAADF tackling of atomic displacements in conductive LAO/STO was based on an image, which exhibits both cation and oxygen columns[8]. However, oxygen columns should be dark in the HAADF imaging due to the weak scattering power of oxygen compared to the significant ones of cations in such oxides[9]. The simultaneous appearance of oxygen and cation columns in the HAADF image[8] could be the consequence of a compromised electron-optics condition employed, rendering certain uncertainties on the reported cation and oxygen

displacements. In addition, the STEM report[8] was not aware of the characteristic oxygen-cage rotations in LAO/STO (Supplementary Note 2 below). The argued structural aspects in Ref. 8 may be disregarded, accordingly.

**Supplementary Note 2. Oxygen-cage rotations and hidden lattice instabilities.** The *ab initio* calculations were performed on the basis of density functional theory with the generalized gradient approximation (GGA)[10]. The accurate full-potential projector-augmented wave method[11], implemented in the Vienna *ab initio* simulation (VASP) package[12], was used together with a large plane cutoff energy of 450 eV. In the self-consistent electronic structure calculations, the total energy convergence criterion was set to be $10^{-6}$ eV. In the structural optimization calculations, atoms were allowed to relax until the associated forces are smaller than 0.01 eVÅ$^{-1}$.

Firstly, we calculated the lattice parameters of LAO and STO. The tetrahedron method with Blöchl corrections for the Brillouin zone integration with a $\mathbf{\Gamma}$-centered Monkhorst-Pack *k*-point mesh of 20×20×20 was used. The thus-calculated cell dimensions are 3.9394 and 3.8196 Å for STO and LAO, respectively. The lattice mismatch between the calculated LAO and STO, ~3.0%, agrees with the experimental ~2.9%.

The calculations of LAO/STO were then performed using the supercell-slab approach with an asymmetric $(LAO)_6/(STO)_9$ configuration and a $TiO_2$-plane interface as shown in Supplementary Fig. 4. Two neighboring slabs are separated by a vacuum layer of ~12 Å (Supplementary Fig. 4), which is sufficiently thick to avoid overlap of electron wave functions from the two neighboring slabs. Furthermore, we incorporated the dipole corrections along *c*-axis to remove the unphysical dipolar interaction between the two neighboring slabs[13]. The in-plane lattice constant of the $(LAO)_6/(STO)_9$ supercell was fixed to 3.9394 Å of the calculated STO and all constituent atoms were relaxed except for those in the left-most $TiO_2$-SrO-$TiO_2$ layers (Supplementary Fig. 4) in order to mimic the effect of unstrained STO deep into the substrate side as observed in Fig. 2a. The former studies of LAO/STO have pointed out that the constituent oxygen cages should exhibit a continuous rotation across the interface[14]. The rotation pattern in the LAO was further found to be the $a^-a^-c^0$ type in Glazer's notation[15,16], namely an anti-phase rotation in *ab*-plane and no rotation along *c*-axis. Both our calculations (Supplementary Fig. 4) and associated ABF imaging of the oxygen cages (Fig. 2e and Supplementary Fig. 4a) suggest such $a^-a^-c^0$ rotation for the LAO and STO and unveil the rotation angle of ~7~8° (~3~5°) along *a*- and *b*-axes of the LAO (STO), in good agreement with the SXRD report of 8~9° for the LAO in metallic heterostructures[15]. The anti-phase rumpling of oxygen cages results in vanishing oxygen contribution to the FE-like polarizations in LAO and STO both along *c*-axis and in *ab*-plane. The previous SXRD studies of LAO/STO did not reveal such oxygen-cage rotations[5-7] until the recent one dedicated to the study of low-symmetry peaks predominated by the structural factor of oxygen[15], readily revealing the $a^-a^-c^0$-type pattern in the metallic LAO/STO.

We subsequently estimated the epitaxial-strain energy stored in the pseudomorphic-strained LAO by calculating the total energy of LAO as a function of *c*-axis, with *ab*-plane clamped to that of the calculated STO. We obtained the contracted *c*-axis of 3.7345 Å, Young's modulus of 364 GPa (experiment, 306 GPa)[17], and the strain energy of ~0.112$t$ eV$a^{-2}$ ($t$, film thickness in $a$; $a$, 4 Å for convenience). This energy agrees with ~0.136$t$ eV$a^{-2}$ evaluated by the classical pseudomorphic-epitaxy formulation (Supplementary Note 5 later).

Such *c*-dependent calculations were also performed for STO and we obtained the Young's modulus of 339 GPa (experiment, 330 GPa)[18] and, most importantly, the onset of FE-like polarization displacements along *c*-axis, which is particularly obvious upon $c > a$ ($a = a_{STO}$,

Supplementary Fig. 5a) due to the incipient FE instability of STO (refs. 19,20). Intriguingly, the FE instability can appear at *c/a* = ~0.99 (ref. 20; Supplementary Fig. 5a), while quenched in cubic STO (*c/a* = 1) due to quantum fluctuations[19,20]. In Supplementary Fig. 5b, the energy gain (*ΔE*; the total-energy difference between *c*-strained uc with and without polarization displacements) of this FE instability in STO can be implicitly described by $\Delta E = AP^2 + BP^4$, with $A = 3.77 \times 10^{-5}$ and $B = -5.79 \times 10^{-7}$ eVuc$^{-1}$, in Landau-Ginzburg-Devonshire theory of FE phase transitions[21]. Surprisingly, FE-like polarizations along *c*-axis also appear in LAO upon *c/a* > 1.01 (Supplementary Fig. 5a, $a = a_{STO}$; i.e., *ab*-plane clamped by STO, Fig. 2d), with readily-derived $A = 8.80 \times 10^{-7}$ and $B = -4.30 \times 10^{-8}$ eVuc$^{-1}$ (Supplementary Fig. 5b). A FE-like instability apparently exists in such *ab*-plane-stretched, *c*-elongated LAO, while totally unexpected[22,23].

**Supplementary Note 3. Calculations of phonon dispersions in LAO and STO.** A signature of FE instabilities in such perovskite oxides is the existence of a soft-phonon mode at zone-center **Γ** point, which breaks the inversion symmetry along *c*-axis[21,24]. We thus performed extensive calculations of the phonon dispersions in LAO and STO within the density functional perturbation theory[25] with GGA. The ultrasoft pseudopotential plane wave method implemented in the Quantum Espresso program[26] was exploited together with a plane wave cutoff energy of 60 Ry. Supplementary Fig. 6 shows the calculated phonon dispersions in (a) *c*-elongated STO, (b) cubic LAO, and *c*-elongated LAO (c) with and (d) without the *ab*-plane clamped by STO. The theoretically considered *c*/*a* is derived from experiments.

In Supplementary Fig. 6a ($c/a_{STO} = 1.01$), the *c*-elongated STO with a FE-like polarization in Supplementary Fig. 5 is indeed characterized by the corresponding soft phonon at **Γ** point, with the characteristic negative frequency (i.e., purely imaginary) revealing its lattice-instability essence[24]. The real-space illustration of this soft mode constitutes the displacive, cooperative B-site polarization movements along *c*-axis[24]. In addition, we observed a lattice instability in the STO at otherwise zone-corner **R** point, which is associated with the octahedral rotation in the tetragonal phase transition at low temperature[24,27,28]. Such a zone-corner soft mode also exists in LAO (Supplementary Figs. 6b-d) due to the octahedral-rotation instability in the ground-state rhombohedral structure[24,27,28]. Most importantly, the *ab*-plane-stretched, *c*-elongated LAO indeed exhibits the zone-center soft phonon (Supplementary Fig. 6c; $c/a = 1.02$, $a = a_{STO}$), firmly establishing the FE-like instability along *c*-axis previously revealed in Supplementary Fig. 5. In *c*-elongated LAO without the *ab*-plane stretching (Supplementary Fig. 6d; $c/a_{LAO} = 1.02$) and cubic LAO (Supplementary Fig. 6b), this lattice instability at **Γ** point disappears. All these microscopic calculations in Supplementary Figs. 5,6 support the experimental proposal that there exist hidden FE-like instabilities in LAO and STO, resurrected near the interface by epitaxial strain, and these FE-like distortions provide a strain-accommodation alternative.

**Supplementary Note 4. Holes in LAO and band bending below Fermi level in STO.** The holes observed in the LAO of metallic 4-, 5-, and 10-uc LAO/STO (Fig. 1e; Supplementary Fig. 7) are summarized in Table 1. The relatively shorter length scale of the holes, compared to that of the 2DEG (Fig. 1e), could be associated with the smaller relative permittivity *ε* of LAO (24, refs. 29,30; STO ~332, ref. 31), which leads to a shorter characteristic Thomas-Fermi screening length in perovskites[31,32].

In addition, the 2DEG in the STO of metallic heterostructures can result in a band bending below the Fermi level ($_\Delta\xi$), with $n = (8\pi^2 m^* {}_\Delta\xi/h^2)^{3/2}/3\pi^2$ (refs. 32,33), where *n* is the 2DEG

density, $m^*$ ($m$) is the effective (rest) mass of electrons with $m^* \approx 3m$ (ref. 34), and $h$ is the Planck's constant. Using $n$ ~4.4 × $10^{13}$ $cm^{-2}$ in 10-uc LAO/STO, $_\Delta\xi$ attains ~50 meV (Table 1) consistent with the experimental derivation of ~30 meV by cross-sectional scanning tunneling spectroscopy[35].

**Supplementary Note 5. The classical evaluation of the epitaxial strain energy.** The epitaxial-strain energy ($u$) stored in a pseudomorphic-strained film is formulated by $u = [2G(1+\nu)/(1-\nu)]\sigma^2 t$ (refs. 36,37), where $G$ is the shear modulus of the film (LAO, 123.77 GPa)[17], $\nu$ is the Poisson's ratio, $\sigma$ is the misfit strain, and $t$ is the film thickness. The thus-evaluated strain energy amounts to ~0.136$t$ eV$a^{-2}$ (gray histograms, Fig. 3; $a$, 4 Å). Besides, the FE-like distortion energy in Fig. 3 consists of the atomic-buckling and thus-induced electrostatic energies[7,38], with the respective formulations of $Ya(d_A{}^2+d_B{}^2)$ and $e(q_A d_A+q_B d_B)/\varepsilon\varepsilon_0 a^2$, where $Y$ is the Young's modulus (LAO, 306; STO, 330 GPa)[7,17,18] and $a$ is uniformly 4 Å for simplicity.

**Supplementary Note 6. EELS tackling of oxygen vacancies.** The 2DEG formation in STO of metallic LAO/STO has been correlated with oxygen vacancies[6,39] and STEM-EELS studies of the problem have been reported[8,40]. However, the EELS tackling of oxygen content across the LAO/STO interface remains an unsettled problem due to the notably different spectral weights and line shapes between the O $K$-edge spectra of bulk LAO and STO as a result of their distinct electronic structures[8], with the spectral characteristics of the interfacial uc subtly different from those of the parent bulks[8,40]. The estimation of the oxygen content by comparing the integrated O $K$-edge intensities in respective interfacial uc to those of the bulks or fitting the characteristic EELS line shapes with the reference bulk-LAO and -STO spectra would, therefore, incorporate systematic uncertainties[8,40]. We tend to discuss the oxygen content by a comparative study that tackles 10-uc LAO/STO without oxygen post-annealing and first-principles calculations of the projected density of states (DOS) of oxygen in bulk and oxygen-deficient STO (Supplementary Fig. 8).

Supplementary Fig. 8a shows the O $K$-edge spectra of 3-, 4-, 5-, and 10-uc LAO/STO studied in Figs. 1 and 2, and those of the 10-uc LAO/STO without post-annealing (electron density, ~1.1 × $10^{14}$ $cm^{-2}$; Methods). Note that each spectrum in Supplementary Fig. 8a is the sum over 5 EELS datasets and acquired on #–3 STO uc, where the 2DEG in metallic 4-, 5-, and 10-uc LAO/STO shows a maximum (Fig. 1e). For a more extensive comparison, we incorporate the O $K$-edge spectra at #–1 and –2 STO uc in the 10-uc LAO/STO without post-annealing (gray, Supplementary Fig. 8a). Basically, the O $K$-edge spectra of the 3-, 4-, 5-, and 10-uc LAO/STO are similar (Supplementary Fig. 8a), with peak A contributed from Ti-3$d$ $t_{2g}$ states, B from mixtures of Ti-3$d$ $e_g$ and Sr-4$d$, and C from Ti-4$sp$, Sr-4$d$, and Sr-5$sp$ as suggested by the orbital-decomposed projected DOS of bulk STO in Supplementary Fig. 8b (omitting intermixing for simplicity). By contrast, peak A in the spectra of 10-uc LAO/STO without post-annealing is noticeably broader (Supplementary Fig. 8a). Comparing the calculated DOS of bulk and oxygen-deficient STO (Supplementary Fig. 8c), the broadening of peak A is visibly an electronic effect of oxygen vacancies and the reported O $K$-edge spectra of oxygen-deficient STO reveal such a broadening tendency[41]. The 10-uc LAO/STO without post-annealing should host certain electron-donor oxygen vacancies, corroborating the higher 2DEG density found in heterostructures without the vacancy-healing treatment[42]. To shed more light on oxygen vacancies possibly in the metallic 4-, 5-, and 10-uc LAO/STO, we make an assumption that the 2DEG with an average density of ~4 × $10^{13}$ $cm^{-2}$ (Table 1) arises from oxygen vacancies,

corresponding to an oxygen deficiency of ~0.03 vacancy per uc, i.e., ~1% of the stoichiometric oxygen content. Considering the general detection limit of ~2% for STEM-EELS (refs. 8,31,43), such an oxygen deficiency of ~1% would be anyhow within the experimental uncertainty of Supplementary Fig. 8a. Accordingly, the spectral similarity in the insulating 3- and metallic 4-, 5-, and 10-uc LAO/STO (Supplementary Fig. 8a) suggests that the oxygen content in these interfacial STO uc is comparable and close to the nominal stoichiometry without traceable evidence for vacancies.

We are aware of an otherwise oxygen estimation[8] by comparing the intensity interplay between peak A and the low-energy shoulder of peak B in Supplementary Fig. 8a. As shown in Supplementary Fig. 8a, this spectral shoulder is nonetheless broad and diffused and the corresponding oxygen-evaluation method[8] would not be suitable hereby. Trials of integrating background-subtracted intensities of O-*K* spectra within a given spectral range for oxygen-content estimations, as previously proposed[44,45], were unfruitful, because the proximity of Ti-*L* to O-*K* edges renders the O-*K* background subtraction easily influenced by the spectral features related to the Ti-*L* edge, affecting the derived O-*K* signals[46]. Oxygen quantification by spectral integrations may not be generally applicable[47]. The 2DEG in our metallic LAO/STO is irrelevant with oxygen vacancies (Supplementary Fig. 8) and the existence of holes in the LAO uc (Fig. 1e) excludes electron-donor oxygen vacancies therein. The oxygen content in our LAO/STO studied in Figs. 1 and 2 should be close to stoichiometry.

It should be mentioned that the surface LAO uc (#3) in 3-uc LAO/STO is susceptible to beam damage upon the STEM-EELS mapping, while robust to separate fast HAADF imaging and spectral line scans (e.g., Supplementary Figs. 7,8). The surface uc in the 4-, 5-, and 10-uc LAO/STO is robust in all our experiments and this difference may be related to the characteristic strength of the surface uc in the prepared 3-uc STEM specimen. In 3-uc LAO/STO, the stoichiometry estimation of the AO ($BO_2$) plane of the surface uc at #2.5-uc (#3-) position is, therefore, subject to larger errors of ± 20% (Fig. 1c and Supplementary Fig. 1a), with the La curves being properly renormalized with reference to the Sr ones such that the nominal AO-plane stoichiometry of unity[31,43] is preserved at the surface LAO uc. The Sr profiles of 3-uc LAO/STO in Fig. 1c and Supplementary Fig. 1a were derived from the systematic comparison of Sr-integral intensities to that at bulk-like #–5.5 STO-uc position, which is free from intermixing, immune from beam sensitivity, and thus ideal as the internal reference.

**Supplementary Note 7. Al-*K* edge at 1560 eV.** Due to the available channel number of 1340 in our spectrometer, the maximal accessible EELS range would correspond to 1340 eV (dispersion, 1 eV/pixel in maximum). Upon STEM-EELS mapping with the maximal dispersion, the acquisition of the deep Al-*K* edge (1560 eV) is unachievable if the shallowest Sr-$M_3$ edge (269 eV) in LAO/STO is to be simultaneously recorded with proper pre-edge range (~50 eV), which is essential for thorough background examinations. The background subtraction was performed by a careful placement of the background-derivation window (~15 eV) before the edge onset and the subsequent Sr mapping was conducted using an intensity-integration window of 4 eV (refs. 31,43). The tackling of Al-*K* edge requires shifts of the spectrometer range to a deeper energy-loss regime (e.g., Supplementary Fig. 9a, 1 eV/pixel) at the cost of Sr-$M_3$ edge[40]. A simultaneous EELS acquisition of Sr, Ti, O, La, and Al edges is, therefore, impossible in our experimental setup and also in the former one exploiting the same spectrometer in a different STEM apparatus (acceleration voltage, 100 keV)[40].

Despite the above limitation, we recorded Al map using a properly selected energy-loss range as shown in Supplementary Fig. 9a, with the customary STEM-EELS condition of 150 ms per pixel and ~78 pA in probe current. However, the inelastic scattering cross section of Al-*K* edge is too low to be unveiled in the STEM-EELS mapping as shown in the inset (Supplementary Fig. 9a), which stands for the blowup of spectral integration over the indicated LAO area in the simultaneously acquired HAADF (red dashed rectangle). Instead, the Ti map in the STEM-EELS dataset shows atomic resolution (green, Supplementary Fig. 9a). A further increase of probe current to maximal ~136 pA in our STEM did not achieve Al mapping, while damaging the specimen. A prolonged acquisition of the Al-*K* edge in an individual LAO uc using optimized probe current of ~78 pA was unsuccessful (Supplementary Fig. 9b), either. Therefore, we had to exploit the alternative of (1 – Ti) for the Al. While this has been a compromised solution, the confidence level in the STEM-EELS quantification of Ti is high as established in our relevant studies in Refs. 31 and 43, where the existence of localized electron density at an insulating oxide interface[43] and the condensation of two-dimensional oxide-interfacial charges into one-dimensional electron chains[31] were readily derived. The thus-derived (1 – Ti) for the Al would be acceptable.

In Ref. 40, the reported Al mapping was assisted by an enhanced inelastic scattering cross section at the lower acceleration voltage[48] of 100 keV and a notable probe current of hundreds of pA ascribed to higher-order aberration corrections[40,49] than our STEM, by using proper sample thickness and/or optimally short pixel time to reduce beam damage (not specifically documented therein, whereas the 100-keV beam causing less sample damage than our 200-keV one[46,49]). The signal-to-noise level in the Al map[40] is, therefore, not optimal for an unambiguous quantitative estimation, not to mention that the Sr-$M_3$ edge essential for Sr quantifications is missing in the datasets due also to the limit of available EELS channels. We are also aware of the Sr-$L_{2,3}$ edges at 1940 and 2007 eV, which could be an alternative for the Sr mapping[45], while the weak mapping contrasts characteristic to deep excitations would be marginal for quantifications at high accuracy[40].

## Supplementary References


1. Aso, R., Kan, D., Shimakawa, Y. & Kurata, H. Atomic level observation of octahedral distortions at the perovskite oxide heterointerface. *Sci. Rept.*, DOI: 10.1038/srep02214 (2013).
2. Jia, C.-L., Mi, S.-B., Urban, K., Vrejoiu, I., Alexe, M. & Hesse, D. Atomic-scale study of electric dipoles near charged and uncharged domain walls in ferroelectric films. *Nat. Mater.* **7**, 57-61 (2008).
3. Galindo, P. L. *et al*. The peak pairs algorithm for strain mapping from HRTEM images. *Ultramicroscopy* **107**, 1186-1193 (2007).
4. Ishizuka, K., HREM Research Inc., http://www.hremresearch.com/ (2015).
5. Willmott, P. R. *et al*. Structural basis for the conducting interface between $LaAlO_3$ and $SrTiO_3$. *Phys. Rev. Lett.* **99**, 155502 (2007).
6. Yamamoto, R. *et al*. Structural comparison of *n*-type and *p*-type $LaAlO_3/SrTiO_3$ interfaces. *Phys. Rev. Lett.* **106**, 036104 (2011).
7. Pauli, S. A. *et al*. Evolution of the interfacial structure of $LaAlO_3$ on $SrTiO_3$. *Phys. Rev. Lett.* **106**, 036101 (2011).
8. Cantoni, C. *et al*. Electron transfer and ionic displacements at the origin of the 2D electron gas at the LAO/STO interface: direct measurements with atomic-column spatial resolution.

*Adv. Mater.* **24**, 3952-3957 (2012).
9. Kim, Y.-M. *et al*. Y. Probing oxygen vacancy concentration and homogeneity in solid-oxide fuel-cell cathode materials on the subunit-cell level. *Nat. Mater.* **11**, 888-894 (2012).
10. Perdew, J. P., *Electronic Structure of Solids '91* 11 (edited by Ziesche, P. & Eschrig, H.; Akademie Verlag, Berlin, 1991).
11. Blöchl, P. E. Projector augmented-wave method. *Phys. Rev. B* **50**, 17953-17979 (1994).
12. The VASP team, https://www.vasp.at/ (2009).
13. Makov, G. & Payne, M. C. Periodic boundary conditions in *ab initio* calculations. *Phys. Rev. B* **51**, 4014-4022 (1995).
14. Ganguli, N. & Kelly, P. J. Tuning ferromagnetism at interfaces between insulating perovskite oxides. *Phys. Rev. Lett.* **113**, 127201 (2014).
15. Johnson-Wilke, R. L. *et al*. Quantification of octahedral rotations in strained $LaAlO_3$ films via synchrotron x-ray diffraction. *Phys. Rev. B* **88**, 174101 (2013).
16. Hatt, A. J. & Spaldin, N. A. Structural phases of strained $LaAlO_3$ driven by octahedral tilt instabilities. *Phys. Rev. B* **82**, 195402 (2010).
17. Luo, X. & Wang, B. Structural and elastic properties of $LaAlO_3$ from first-principles calculations. *J. Appl. Phys.* **104**, 073518 (2008).
18. Hachemi, A., Hachemi, H., Ferhat-Hamida, A. & Louail, L. Elasticity of $SrTiO_3$ perovskite under high pressure in cubic, tetragonal and orthorhombic phases. *Phys. Scr.* **82**, 025602 (2010).
19. Müller, K. A. & Burkard, H. $SrTiO_3$: An intrinsic quantum paraelectric below 4 K. *Phys. Rev. B* **19**, 3593-3602 (1979).
20. Lin, C.-H., Huang, C.-M. & Guo, G. Y. Systematic *ab initio* study of the phase diagram of epitaxially strained $SrTiO_3$. *J. Appl. Phys.* **100**, 084104 (2006).
21. Fridkin, V. & Ducharme, S. *Ferroelectricity at the Nanoscale* 1-10 (Springer-Verlag, Berlin, Heidelberg, 2014).
22. Bark, C. W. *et al*. Switchable induced polarization in $LaAlO_3/SrTiO_3$ heterostructures. *Nano Lett.* **12**, 1765-1771 (2012).
23. Sharma, P. *et al*. Mechanical tuning of $LaAlO_3/SrTiO_3$ interface conductivity. *Nano Lett.* **15**, 3547-3551 (2015).
24. Ghosez, Ph., Cockayne, E., Waghmare, U. V. & Rabe, K. M. Lattice dynamics of $BaTiO_3$, $PbTiO_3$, and $PbZrO_3$: A comparative first-principles study. *Phys. Rev. B* **60**, 836-843 (1999).
25. Giannozzi, P., de Gironcoli, S., Pavone, P. & Baroni, S. *Ab initio* calculation of phonon dispersions in semiconductors. *Phys. Rev. B* **43**, 7231-7242 (1991).
26. The Quantum Espresso project, http://www.quantum-espresso.org/ (2015).
27. Müller, K. A., Berlinger, W. & Waldner, F. Characteristic structural phase transition in perovskite-type compounds. *Phys. Rev. Lett.* **21**, 814-817 (1968).
28. Axe, J. D., Shirane, G. & Müller, K. A. Zone-boundary phonon instability in cubic $LaAlO_3$. *Phys. Rev.* **183**, 820-823 (1969).
29. Cancellieri, C. *et al*. Electrostriction at the $LaAlO_3/SrTiO_3$ interface. *Phys. Rev. Lett.* **107**, 056102 (2011).
30. Bark, C. W. *et al*. Tailoring a two-dimensional electron gas at the $LaAlO_3/SrTiO_3$ (001) interface by epitaxial strain. *PNAS* **108**, 4720-4724 (2011).
31. Chang, C. P. *et al*. Condensation of two-dimensional oxide-interfacial charges into one-dimensional electron chains by the misfit-dislocation strain field. *Nat. Commun.*, DOI: 10.1038/ncomms4522 (2014).

32. Copie, O. *et al*. Towards two-dimensional metallic behavior at $LaAlO_3/SrTiO_3$ interfaces. *Phys. Rev. Lett.* **102**, 216804 (2009).
33. Sluka, T., Tagantsev, A. K., Damjanovic, D., Gureev, M. & Setter, N. Enhanced electromechanical response of ferroelectrics due to charged domain walls. *Nat. Commun.*, DOI: 10.1038/ncomms1751 (2012).
34. Mannhart, J. & Schlom, D. G. Oxide interfaces – an opportunity for electronics. *Science* **327**, 1607-1611 (2010).
35. Huang, B.-C. *et al*. Mapping band alignment across complex oxide heterointerfaces. *Phys. Rev. Lett.* **109**, 246807 (2012).
36. Müller, P & Kern, R. Some elastic effects in crystal growth, in *Stress and Strain in Epitaxy* 3-61 (edited by Hanbücken, M. & Deville, J.-P.; Elsevier Science, Amsterdam, 2001).
37. Matthews, J. W. Defects associated with the accommodation of misfit between crystals. *J. Vac. Sci. Technol.* **12**, 126-133 (1975).
38. Gureev, M. Y., Tagantsev, A. K. & Setter, N. Head-to-Head and tail-to-tail 180° domain walls in an isolated ferroelectric. *Phys. Rev. B* **83**, 184104 (2011).
39. Yoshimatsu, K., Yasuhara, R., Kumigashira, H. & Oshima, M. Origin of metallic states at the heterointerface between the band insulators $LaAlO_3$ and $SrTiO_3$. *Phys. Rev. Lett.* **101**, 026802 (2008).
40. Warusawithana, M. P. *et al*. $LaAlO_3$ stoichiometry is key to electron liquid formation at $LaAlO_3/SrTiO_3$ interfaces. *Nat. Commun.*, DOI: 10.1038/ncomms3351 (2013).
41. Muller, D. A., Nakagawa, N., Ohtomo, A., Grazul, J. L. & Hwang, H. Y. Atomic-scale imaging of nanoengineered oxygen vacancy profiles in $SrTiO_3$. *Nature* **430**, 657-661 (2004).
42. Cen, C. *et al*. Nanoscale control of an interfacial metal-insulator transition at room temperature. *Nat. Mater.* **7**, 298-302 (2008).
43. Chang, C. P. *et al*. Atomic-scale observation of a graded polar discontinuity and a localized two-dimensional electron density at an insulating oxide interface. *Phys. Rev. B* **87**, 075129 (2013).
44. Nakagawa, N., Hwang, H. Y. & Muller, D. A. Why some interfaces cannot be sharp. *Nat. Mater.* **5**, 204-209 (2006).
45. Gauquelin, N. *et al*. Atomically resolved EELS mapping of the interfacial structure of epitaxially strained $LaNiO_3/LaAlO_3$ superlattices. *Phys. Rev. B* **90**, 195140 (2014).
46. Botton, G. A., Lazar, S. & Dwyer, C. Elemental mapping at the atomic scale using low accelerating voltages. *Ultramicroscopy* **110**, 926-934 (2010).
47. Nücker, N. *et al*. Site-specific and doping-dependent electronic structure of $YBa_2Cu_3O_x$ probed by O 1*s* and Cu 2*p* x-ray-absorption spectroscopy. *Phys. Rev. B* **51**, 8529-8542 (1995).
48. Raether, H. *Excitations of plasmons and interband transitions by electrons* 1-64 (Springer-Verlag, Berlin, Heidelberg, 1980).
49. Muller, D. A. *et al*. Atomic-scale chemical imaging of composition and bonding by aberration-corrected microscopy. *Science* **319**, 1073-1076 (2008).
